# Impacts of COVID-19 on Electronic Cigarette Purchasing, Use and Related Behaviors

**DOI:** 10.3390/ijerph17186762

**Published:** 2020-09-16

**Authors:** Eric K. Soule, Shannon Mayne, William Snipes, Mignonne C. Guy, Alison Breland, Pebbles Fagan

**Affiliations:** 1 Department of Health Education and Promotion, College of Health and Human Performance East Carolina University, Greenville, NC 27858, USA; maynes20@ecu.edu (S.M.); snipesw17@students.ecu.edu (W.S.); 2 Center for the Study of Tobacco Products, Virginia Commonwealth University, Richmond, VA 23220, USA; mguy@vcu.edu (M.C.G.); abbrelan@vcu.edu (A.B.); pfagan@uams.edu (P.F.); 3 Department of African American Studies, College of Humanities and Sciences, Virginia Commonwealth University, Richmond, VA 23220, USA; 4 Department of Psychology, College of Humanities and Sciences, Virginia Commonwealth University, Richmond, VA 23220, USA; 5 Center for the Study of Tobacco, Department of Health Behavior and Health Education, Fay W. Boozman College of Public Health, University of Arkansas for Medical Sciences, Little Rock, AR 72205, USA

**Keywords:** electronic cigarettes, COVID-19, tobacco

## Abstract

*Background*: COVID-19 has caused health impacts and disruptions globally. Electronic cigarette (ECIG) users may face additional impacts. This study examined impacts of COVID-19 on ECIG users. *Methods*: Concept mapping, a mixed-methods approach, was used to identify COVID-19 impacts on adult ECIG users. ECIG users (*n* = 93) provided statements completing a prompt: “A specific way Coronavirus/COVID-19 has affected my vaping/e-cigarette use, my vaping/e-cigarette related purchasing, or other vaping/e-cigarette related behaviors or issues is…”. Participants generated 85 unique statements, sorted statements into groups of similar content and rated each statement on how true they were. Multidimensional scaling and hierarchical cluster analysis identified thematic clusters. Mean cluster ratings were compared between sample subgroups. *Results*: Ten clusters were identified: Stocking up and Bulk Purchasing, Challenges in Obtaining ECIG Supplies, Alternative Purchasing Procedures, Increased ECIG use, Disruption of Routine and ECIG Use, Efforts to Decrease ECIG Use, Improving ECIG Skills, COVID-19 Health Concerns, Perceptions of ECIG Use and COVID-19, and COVID-19 Protection. More dependent ECIG users and dual users of ECIGs and cigarettes rated clusters higher than less dependent ECIG users and non-dual users. *Conclusions:* ECIG users may experience or perceive they face additional COVID-19 impacts, such as increased exposure, financial burdens, stress, and health risks.

## 1. Introduction

In 2020, COVID-19, a disease caused by a novel coronavirus (SARS-CoV-2), spread rapidly across the world in all continents, with the exception of Antarctica [1]. In September 2020, over 29,110,000 COVID-19 cases and over 925,000 deaths attributed to the disease were reported worldwide [1], including more than 6,500,000 COVID-19 cases and 193,000 deaths reported in the United States, the country with the highest number of COVID-19 cases and deaths as of September 2020 [2].

Health organizations and government bodies and officials have recommended preventative measures including limiting physical contact, such as shaking hands or hugging others, and keeping at least six feet of space between oneself and others, commonly referred to as “social distancing” [3]. To promote social distancing, almost all states in the United States implemented some type of “stay at home,” “safer at home,” or “shelter in place” order [4] in which residents are required or recommended to stay home with minimal exceptions, large gatherings of people are prohibited or advised against, and many businesses are temporarily closed or operate at reduced capacity. 

The known health risks, recommendations and stay at home orders that have been implemented to prevent the spread of COVID-19, and stressors associated with the pandemic have had major impacts on daily life for many people around the world. Little is known about the physical, psychological, emotional, financial, and other types of impacts the COVID-19 pandemic and stay at home orders have had on the population. One population that may feel additional impacts due to disruptions caused by COVID-19 is electronic cigarette (ECIG) users. In the United States, ECIG use has increased in recent years [5,6,7,8,9,10,11,12,13,14]. While great attention has been given to high rates of ECIG use among youth [14], there were an estimated 8.1 million adult current ECIG users in 2018, an increase in prevalence from 2.8% to 3.2% during 2017–2018 [6]. 

Protection Motivation Theory, which has been used to predict smoking behaviors [15,16], may be useful for predicting ECIG use behaviors during the COVID-19 pandemic. Protective Motivation Theory suggests that when faced with a threat, cognitive appraisals of threat and coping influence healthy or unhealthy behaviors related to the threat (e.g., COVID-19) [17]. Using the threat appraisal pathway, ECIG users may compare the perceived rewards and the perceived threats of the maladaptive behavior (i.e., ECIG use). Using the coping appraisal pathway, ECIG users may compare coping efficacy (e.g., not using ECIGs) with the cost of not using ECIGs. Dependent ECIG users may face additional challenges in obtaining ECIG products due to being required to stay at home or loss of income. Additionally, the stress of COVID-19 may also cause ECIG users to change their ECIG use behaviors. These impacts caused directly by COVID-19 could have potential health impacts on ECIG users. Therefore, the purpose of this study was to examine how the COVID-19 pandemic has impacted ECIG use, ECIG product purchasing, and other related behaviors or issues.

## 2. Materials and Methods 

### 2.1. Overview

This study was approved by the East Carolina University and Medical Center Institutional Review Board and used concept mapping [18], a mixed-methods approach that incorporates participant tasks (brainstorming, sorting, and rating) and qualitative and quantitative data analysis to generate a model that organizes and describes content related to a research topic (i.e., impacts of COVID-19 on ECIG users). These methods are described further below.

### 2.2. Participants

During 2019, a panel of current (past-30 day) ECIG users were recruited by posting advertisements at 24 Craigslist locations selected randomly from each of the four U.S. census regions (Northeast, Midwest, South, West; as in [19,20,21]). Interested individuals completed an online screening questionnaire. In April 2020, individuals over the age of 18 who reported current ECIG use at the time of completing the screening survey were contacted to be invited to the current study. After confirming current ECIG use status, eligible individuals were emailed instructions for participating in the current study including a link to the study website. At the study website (The Concept Systems^®^ Global MAX©), participants (*n* = 93) provided informed consent and completed a brief questionnaire including items assessing ECIG use, ECIG device and liquid characteristics, ECIG dependence (E-Cigarette Dependence Scale [22]), other tobacco use, and demographic characteristics.

### 2.3. Concept Mapping Procedures

After the brief survey, participants completed three concept mapping study tasks (brainstorming, sorting, and rating). Concept mapping generates statements that describe content related to a focus prompt which are then grouped into clusters of statements that organize and describe content related to a research question. This approach has been used previously to identify and describe ECIG-related content themes such as ECIG dependence [21], adverse effects of ECIG use [23], and ECIG positive outcome expectancies [20].

### 2.4. Brainstorming

At the study website (The Concept System^®^ Global MAX™), participants provided statements completing the focus prompt: “A specific way Coronavirus/COVID-19 has affected my vaping/e-cigarette use, my vaping/e-cigarette related purchasing, or other vaping/e-cigarette related behaviors or issues is…”. This prompt was expected to provide robust data on ECIG users’ behaviors associated withCOVID-19, including those related to Protection Motivation Theory. Participants were encouraged to provide multiple statements. Generated statements were added to an ongoing list. While participants completed this task individually, all statements provided by prior participants were visible to subsequent participants. Participants were instructed to review previous statements when entering their own to avoid providing duplicate content. This process prevents interference due to waiting for one’s turn to provide a statement [24] and allows for interactive brainstorming that generates more [25,26] and unique [27,28] ideas. Statements were reviewed continuously during the brainstorming phase and after content saturation was reached, a final reminder email was sent to eligible individuals to complete the study and the brainstorming task was closed by study staff. Ninety-three participants generated 216 statements in the brainstorming task and each participant received a $10 e-gift card.

Three researchers reviewed the list of statements independently to identify redundant content (e.g., “Seems to be using it more often since bored” and “I do it more because I am bored”) and statements that did not relate to the focus prompt (e.g., “There is no such thing as good vaping and or smoking”) to be removed from the list. If two or more reviewers identified a statement as not relating to the prompt, the statement was removed. If two or more reviewers identified statements as being redundant, one statement that best described the idea using the fewest words possible was retained and all other redundant statements were removed. After review, 85 statements were retained. Reviewers edited the content for consistency (e.g., referring to ECIG use as “vaping” throughout) as well as spelling and grammatical errors. 

### 2.5. Sorting

Participants were then sent an email inviting them to return to the study website to complete the sorting and rating study tasks. For sorting, participants were asked to group (on their own) each of the final 85 statements into “piles” of similar content with instructions: piles had to relate to a single idea based on content similarity and there could not be an “other/miscellaneous” pile or a single pile with all statements. The research team reviewed participants’ sorting tasks and provided feedback to those who did not follow the instructions. After review, the research team approved 77 participants’ sorting activities, well beyond the number of sorts needed to achieve good model fit as identified in a pooled analysis of concept mapping studies [18]. Participants received a $25 e-gift card for sorting statements.

### 2.6. Rating

Participants were asked to rate statements based on the prompt, “This is a way Coronavirus/COVID-19 has impacted my vaping/e-cigarette use, my vaping/e-cigarette related purchasing, or other vaping/e-cigarette related behaviors or issues.” Response options ranged from “1—Definitely NOT true for me” to “7—Definitely true for me.” Eighty-one participants received a $10 e-gift card for completing rating.

### 2.7. Representation

Participant sorting data was used to create an 85 x 85 matrix of similarities such that each cell represented the count for how many times two statements were sorted into the same pile by participants. For example, a “60” in the cell corresponding to statement 2 (“I purchased multiple vaping products because I am uncertain if vaping products will be available in the future”) and statement 5 (“I am purchasing several vaping products at once because I am uncertain if I can leave my home”) indicated these statements were sorted into the same pile by 60 participants in the sorting task. Using software built into the study website, nonmetric multidimensional scaling (MDS) analysis was used to generate a “point map” where each statement was assigned a point in two-dimensional space. The location of the points on the map was determined by an algorithm [29] such that points that were closer together on the map represented statements that were sorted into the same piles by more participants and thus represented more similar content. The stress value of the MDS analysis (an indicator of model fit) was 0.22, within the range of values reported by previous concept mapping studies [18] indicating good model fit and congruence between scaled and raw sorting data.

### 2.8. Analysis

**Figure 1:**
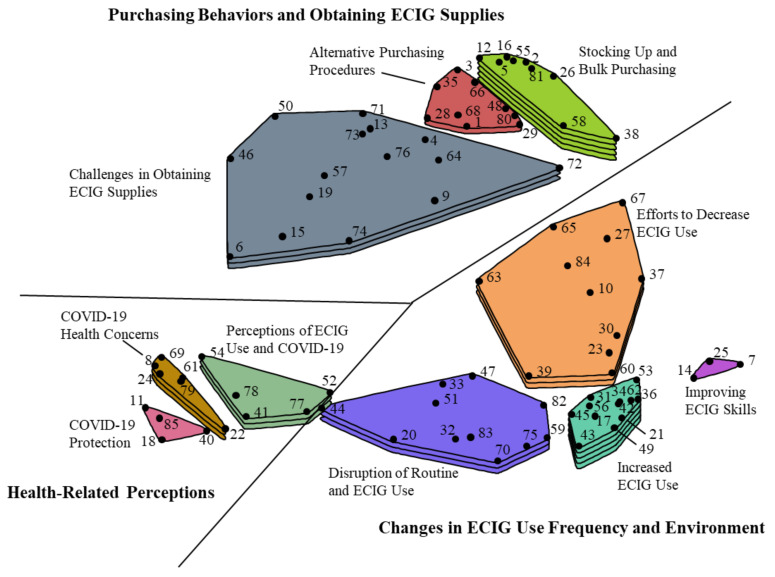
Concept map displaying 10 clusters ECIG user-identified statements describing the impacts of COVID-19 on vaping. Numbered points on the map that are closer to one another represent statements of more similar content whereas points on the map that are further apart represent statements of less similar content. Greater number of layers in clusters indicate higher mean ratings of statements within each cluster based on the rating task. Mean ratings for clusters with 1 layer range from 2.84 to 3.10, 2 layers from 3.10 to 3.36, 3 layers from 3.36 to 3.61, 4 layers from 3.61 to 3.87, and 5 layers from 3.87 to 4.13.

Using an algorithm [30] that identified non-overlapping “clusters” of statements by identifying groups of statements that limited the distance from points to the centroid of identified clusters, a hierarchical cluster analysis examined multiple models. Specifically, concept mapping software first identified quantitatively a two-cluster model. Subsequent models were built by separating one cluster from the previous model into two clusters, similarly identifying clusters by limiting the distance between points and the centroid of the cluster. The goal of this process is to identify the most parsimonious model (i.e., fewest clusters preferred) in which each cluster only relates to a single theme. The research team continued the hierarchical cluster analysis process and through group discussion examined if models met interpretability (i.e., each cluster described a single them) and parsimony (i.e., model with the fewest clusters preferred) criteria. The team determined that the best fitting model was achieved with 10 clusters (see Figure 1). Mean cluster ratings were calculated by taking the average of ratings from all participants for all statements within a single cluster. After confirming rating data met assumptions for statistical tests, mean cluster ratings were compared between participant subgroups based on responses to ECIG use, other tobacco use, and demographic items using Whelch’s independent sample t-tests and an alpha level of 0.05. 

## 3. Results

### 3.1. Participant Characteristics

Sample characteristics are displayed in Table 1. Over half (54.8%) of participants were women and 43.0% were men. Most participants (86.0%) were non-Hispanic and the most common race reported was white (76.3%) followed by black (10.8%) and Asian (6.5%). The average age of participants was 35.1 years old (SD = 10.8). More than half (58.1%) reported using ECIGs every day and approximately three-quarters (76.5%) had been using ECIGs regularly for more than one year. The most common ECIG device type used was a “pod mod such as JUUL” (36.7%) followed by “Rebuildable/Mechanical Mod or Box Mod” (20.4%) and “prefilled disposable/cig-alike” (18.4%). While 80.6% reported that they used ECIG liquid with a nicotine concentration in the range of 1–20 mg/mL, this number may not reflect the actual nicotine concentrations of participants’ ECIG liquid given that the most commonly used device was a JUUL device which has uses pods containing ECIG liquid of higher nicotine concentration: either approximately 59 mg/mL or 35 mg/mL according to the manufacturer website [31] or higher based on results from previous research [32]. The most common locations for purchasing ECIG liquid was at vape shops/tobacco stores (61.3%) or online/over the internet (41.2%). Mean E-Cigarette Dependence Scale [22] score was 2.06 (SD = 0.83) out of a possible 4 (higher scores indicate greater dependence). Just over half (53.8%) were current cigarette smokers and 12.9% had smoked less than 100 cigarettes in their lifetime.

### 3.2. Concept Mapping Results

Ten clusters were identified and organized in a cluster map (see Figure 1) that described themes related to how COVID-19 had impacted ECIG users’ behaviors, perceptions, and emotions. These clusters grouped into three broad themes: Purchasing Behaviors and Obtaining ECIG Supplies, Changes in ECIG Use Frequency and Environment, and Health-Related Perceptions. Statements and clusters on the map that are closer to one another represent more similar content. A summary of the clusters is presented below. A complete list of clusters and statements, including mean cluster and statement ratings, are displayed in Table 2. These clusters are discussed in groups based on content similarity.

#### 3.2.1. Purchasing Behaviors and Obtaining ECIG Supplies

The first group of clusters included statements that related to how COVID-19 had impacted ECIG users’ ability to obtain various ECIG supplies needed for ECIG use. The highest rated cluster in this group of clusters, indicating statements within this cluster were rated as most true for participants, was the *Stocking Up and Bulk Purchasing* cluster. This cluster had a mean cluster rating of 4.13 (SD = 0.61). The nine statements within this cluster described how ECIG users responded to COVID-19 by purchasing greater quantities of ECIG supplies per purchase, such as ECIG liquids or pods, in order to ensure that they did not run out, as in the statement, “I bought extra vaping supplies and e-liquid/pods to stock up.” Statements indicated that ECIG users may be more conscious of their ECIG supplies due to the uncertainty of being able to purchase products if they run out or if products will be available (e.g., “I check my supplies to make sure I have what I need” and “I purchased multiple vaping products because I am uncertain if they will be available in the future”). Some statements also indicated that these bulk purchasing and potential hoarding behaviors may relate to concerns about not being able to leave home due to stay-at-home orders or in attempts to minimize trips to the store.

The second highest rated cluster in this group was the *Challenges in Obtaining ECIG Supplies* cluster (*n*= 15, M = 3.40, SD = 0.94). The statements in this cluster described the numerous types of challenges (or lack thereof) ECIG users faced regarding obtaining ECIG supplies. The highest rated statement in the cluster was “I go to the store less due to social distancing” (M = 5.54), suggesting ECIG users found obtaining ECIG supplies more difficult while also adhering to recommended guidelines for preventing the spread of COVID-19. This was also demonstrated in the statement, “I am hesitant to go out and get more e-liquid/pods because I am scared to go near other people.” Several statements included references to price noting that ECIG users were more aware of prices of ECIG products and some noticed prices had increased during the COVID-19 pandemic. These issues appeared to be related to other statements in the cluster indicating COVID-19 had impacted ECIG users’ income, such as the statement “I am low on money and don’t want to have to spend it on vaping products.) Other statements noted the challenges in returning defective products, that some vape shops were closed, and some participants had used “burnt” pods or other defective ECIG products because obtaining replacement products was challenging. Two statements described no challenges of obtaining ECIG liquid because these ECIG users either made their own ECIG liquid or had someone else make their ECIG liquid. However, these were two of the lowest rated (i.e., least true) of all statements.

The last cluster in this group of clusters related to obtaining ECIG supplies was the *Alternative Purchasing Procedures* cluster (*n* = 9, M = 3.27, SD = 0.82). Statements in this cluster, located in between the *Stocking Up and Bulk Purchasing* and the *Challenges in Obtaining ECIG Supplies* clusters in Figure 1, described new behaviors and procedures ECIG users implemented to obtain ECIG supplies. Some statements described accessing alternative sources to purchase ECIG supplies, such as purchasing products online, going to convenience stores or grocery stores, or purchasing ECIG products from vape shops using “curbside pickup.” Statements suggested that supplies or selections for ECIG products were limited compared to pre-COVID-19 pandemic necessitating ECIG users to call stores ahead of time to evaluate product availability, select from limited options, or purchase an undesirable product because these were the only products available or accessible.

#### 3.2.2. Changes in ECIG Use Frequency and Environment

The next group of four clusters included statements relating broadly to changes in ECIG use behaviors and the locations people engaged in ECIG use due to COVID-19. The highest rated cluster within this group (indicating that participats rated statements in this cluster as being more true) and second highest rated cluster overall was the *Increased ECIG Use* cluster (*n* = 12, M = 4.07, SD = 0.61). While one statement in this cluster indicated a general increase in ECIG use since COVID-19 (“I vape more than usual.”), other statements provided specific reasons for increased ECIG use. Several statements suggested ECIG users were spending more time in locations where ECIG use was allowed, especially at home. The second highest rated statement (M = 4.79) was “I vape more because I am bored” and was consistent with other statements related to having additional free time due to staying at home, not working as much, staying up later at night, and vaping more to cope with being isolated from friends. Several statements also noted attempts to vape more rather than smoke cigarettes.

The next highest rated cluster in this group was the *Disruption of Routine and ECIG Use* cluster (*n* = 11, M = 3.59, SD = 0.96). This cluster included a diverse group of statements, however, they all centered on how COVID-19 had caused significant changes in ECIG users’ daily lives resulting in stress, conflict, and anxiety that ECIG users attempted to reduce. For example, ECIG users reported changes in how they vaped, such as not sharing their ECIG device with others and distancing themselves further from others when vaping. Other statements described more frequent and intense cravings, increased anxiety directly caused by COVID-19, and not feeling well due to a perceived limited ability to vape. Related to the statements from the *Increased ECIG Use* cluster regarding ECIG use in the home, some statements described that ECIG users were more aware of the high frequency of their ECIG use due to being around family members or roommates. For some ECIG users, this resulted in arguments and other statements noted attempts by family members/roommates to prohibit ECIG use in the house.

In between the *Increased ECIG Use* cluster and cluster related to *Challenges in Obtaining ECIG Supplies* clusters (Figure 1) was the *Efforts to Decrease ECIG Use* cluster (*n* = 11, M = 3.38, SD = 0.62). While the statements within this cluster related broadly to ECIG users’ attempts to decrease their ECIG use, the statements made clear these were mostly an attempt to make ECIG supplies last longer rather than attempts to quit or reduce ECIG use. For example, statements included “I am trying to ration my e-liquid/pods” (M = 4.06), “I am more mindful of the amount I vape because it is no longer easy to obtain products in stores or online,” (M = 3.65), and “I am vaping less to try to extend what I have instead of buying more online” (M = 3.38). Other statements noted waiting longer to vape after waking and longer intervals between use. The highest rated statement in the cluster (M = 4.51) described ECIG users vaping tanks/pods “until the very last drop” to prevent going out as often.

The final cluster in this group of clusters, *Improving ECIG Skills*, related to mastery of ECIG-specific behaviors in the additional time that was afforded from staying at home (*n* = 3, M = 2.90, SD = 0.31). These behaviors included trying new flavors and brands, “perfecting” homemade/do-it-yourself (DIY) ECIG liquid recipes, and learning new vape tricks.

#### 3.2.3. Health-related Perceptions

The final group of clusters included three clusters of statements that broadly related to ECIG users’ perceptions of health risk from COVID-19, if ECIG use may affect this risk, and possible actions that may be taken to address this risk. The highest rated cluster in this group was the COVID-19 *Health Concerns* cluster (*n* = 6, M = 3.53, SD = 0.59). The statements in this cluster described how some ECIG users were worried that ECIG use may negatively affect their health, particularly related to COVID-19 risk. While the highest rated statement was more general (“I worry about how vaping is affecting my health.”; M = 4.42), other statements described ECIG users’ concerns and awareness of their own chest pain, lung pain, and cough that may have been associated with ECIG use and could increase the chances of complications from COVID-19. Indeed, responses to the brief survey at the study website indicated many participants attributed adverse health effect symptoms to their ECIG use including cough (31.2%), shortness of breath (19.4%), chest pain (14.0%), and nausea (11.8%). One statement in the cluster suggested some ECIG users had attempted to quit ECIG use until the COVID-19 pandemic had subsided, but this was the lowest rated statement in the cluster (M = 2.52).

The next highest rated cluster in this group was the *Perceptions of ECIG Use and COVID-19* cluster (*n* = 5, M = 3.49, SD = 0.46). The statements in this cluster described perceptions related to COVID-19 and how it may interact with ECIG use. The two highest rated statements in the cluster indicated that some participants were “not scared or concerned about vaping due to COVID-19” (M = 3.91) and “COVID-19 has not affected…vaping at all” (M = 3.88). One statement suggested some ECIG users felt that vaping may have kept them safe due to others avoiding them while vaping. The remaining statements described ECIG users considering quitting ECIG use due to COVID-19, but not necessarily committing to it or ultimately being unsuccessful at quitting.

The final cluster was the *COVID-19 Protection* cluster (*n* = 4, M = 2.84, SD = 0.77). This was the lowest rated (i.e., rated as least true) of all clusters and included statements describing perceptions of being at low risk for COVID-19. While one statement, “I don’t stress too much because I am pretty healthy” (M = 4.17), attributed low risk for COVID-19 because of perceptions of overall good health, the three other statements described perceptions that ECIG use may protect ECIG users from COVID-19. Specifically, statements included “I have wondered if vaping provides a protective layer to prevent COVID-19” (M = 2.53), “I think vaping may kill the COVID-19 virus due to the heat from vaping” (M = 2.33), and “I think vaping will increase my immune system” (M = 2.32).

### 3.3. Cluster Comparisons

**Figure 2:**
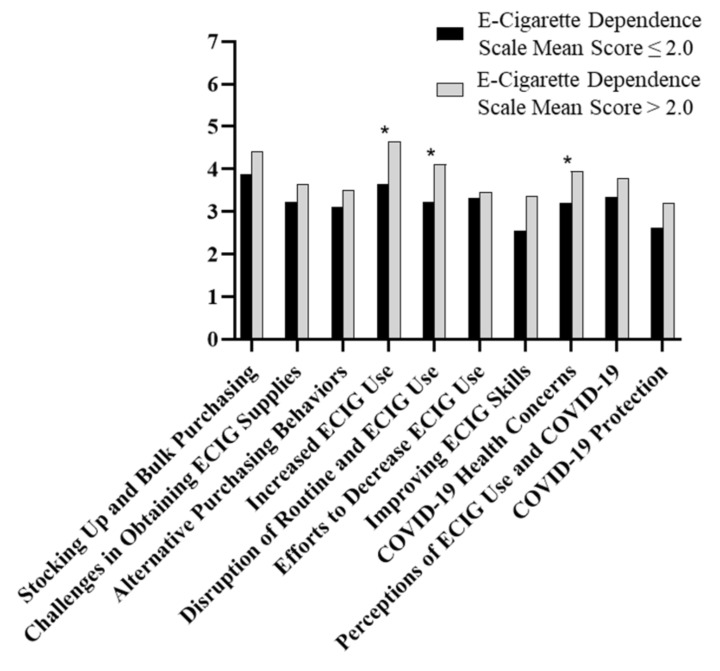
Mean cluster ratings among participants with E-Cigarette Dependence Scale [18] mean scores ≤ 2.0 and > 2.0. * indicates significant difference (*p* < 0.05).

Mean cluster ratings were associated with several ECIG user characteristics. As displayed in Figure 2, ECIG dependence, as assessed by the E-Cigarette Dependence Scale [21], was associated with differences in mean cluster ratings. Participants with mean E-Cigarette Dependence Scale scores above the median score (e.g., ≥2.0) had significantly higher mean cluster ratings than those scores below the median score (e.g., <2.0) on the *COVID-19 Health Concerns* cluster (M = 3.86 vs. M = 3.06; < 0.05), *Increased ECIG Use* cluster (M = 4.62 vs. M = 3.35; *p* < 0.001), and *Disruption in Routine and ECIG Use* cluster (M = 3.99 vs. 3.09; *p* < 0.05). Daily ECIG users had significantly lower ratings for the *Efforts to Decrease ECIG Use* cluster (M = 3.10, SD = 0.63) compared to non-daily ECIG users (M = 3.79, SD = 0.61; *p* < 0.02). There were no differences in mean cluster ratings based on ECIG use frequency, regular ECIG device type used, or where ECIG users typically purchased ECIG liquids before the COVID-19 pandemic. 

ECIG users who also reported current cigarette use (i.e., dual-users) had higher mean cluster ratings than non-current cigarette smokers for the *Stocking Up and Bulk Purchasing* cluster (M = 4.44 vs. 3.69; *p* < 0.05), *Efforts to Decrease ECIG Use* cluster (M = 3.71 vs. M = 3.02; *p* < 0.05), *Improving ECIG Skills* cluster (M = 3.48 vs. M = 2.27; *p* < 0.05), *Increased ECIG Use* cluster (M = 4.34 vs. M = 3.72; *p* < 0.05), and *Disruption in Routine and ECIG Use* cluster (M = 4.07 vs. M = 3.04; *p* < 0.05).

## 4. Discussion

This study identified broad themes describing how the COVID-19 pandemic has impacted ECIG users in the United States. Some impacts related directly to challenges in obtaining ECIG supplies and strategies used to circumvent these challenges. ECIG users also reported attempts to decrease or ration their ECIG use during a time when leaving the home was advised against or supplies were difficult to obtain. However, ECIG users reported increased ECIG use and bulk purchasing of ECIG supplies. For some ECIG users, ECIG use may have caused additional stressors in the form of needing to confront family/roommates about their ECIG use frequency, making decisions about spending money on ECIG supplies when income decreased, putting oneself at risk due to making additional trips to stores to obtain ECIG supplies, or experiencing anxiety about the potential risk for COVID-19 health consequences due to ECIG use. Many of the statements provided by ECIG users in this study described ECIG use as a maladaptive behavioral response to a threat appraisal of COVID-19, such as increased ECIG use or purchasing additional ECIG supplies. More dependent ECIG users as well as dual users of ECIGs and cigarettes appeared to experience these impacts to a greater extent than less dependent ECIG users or non-dual users of ECIGs and cigarettes. 

Statements describing challenges obtaining ECIG products by participants are consistent with many non-essential businesses being required to temporarily close during the COVID-19 pandemic. Indeed, before COVID-19, nearly two thirds of the participants reported getting ECIG supplies from a brick and mortar vape shop or tobacco shop. However, unlike many other drug delivery devices or recreation drugs, ECIG products can be purchased over the internet. Just under half of participants reported purchasing ECIG products online before COVID-19 and statements suggested others shifted to this practice during the COVID-19 pandemic. This availability may pose additional financial risks for ECIG users. That is, this study shows that some ECIG users may continue to purchase ECIG products during times of financial insecurity and some may be willing to take additional risks, such as making additional trips to stores during stay at home orders. These actions demonstrate in novel ways the strength of ECIG dependence and point out the high accessibility of ECIG products. 

Some clusters and statements demonstrated that the COVID-19 pandemic may increase the potential for negative health outcomes for ECIG users. First, the highest rated clusters suggested many ECIG users increased their ECIG use during the COVID-19 pandemic. Thus, many ECIG users are having increased exposure to ECIG aerosol that is associated with known and unknown health effects. Statements describing how some ECIG users are vaping their ECIG devices/pods to “the very last drop” may mean that ECIG users are more likely to puff on an ECIG that has little or no ECIG liquid in contact with the heating element. Research has shown that aerosol generated during conditions where ECIG liquid begins to run out in an ECIG device contains higher amounts of toxicants including aldehydes and carbonyls [33,34]. If ECIG users try to extend their ECIG use without purchasing additional liquid/pods and puff on ECIGs with small amounts of liquid, some ECIG users be exposed to aerosol that is more toxicant laden. This phenomenon may be more common during the COVID-19 pandemic. 

Of greater concern is that some statements indicated ECIG users felt ECIG use may be protective for COVID-19. Indeed, while very preliminary results [35] from data of uncertain quality [36,37] have suggested that nicotine may play a protective role for COVID-19, more research is needed on this topic. However, ECIG use, with and without nicotine, has been shown to disrupt lung surfactant homeostasis [38] which may increase risk for COVID-19 infection and adverse outcomes [39]. Until a time when the relationship between ECIG use and COVID-19 is better understood, physicians should continue to follow Centers for Disease Control guidelines regarding ECIG use: ECIGs may benefit some cigarette smokers looking to quit cigarettes, however, ECIGs are not safe for youth, young adults, pregnant women, or non-tobacco users [40].

This study had several limitations. While the statements described the broad range of impacts COVID-19 has had on ECIG users, the current approach does not allow for prevalence estimates of each of these impacts. This study only examined adults and there may be additional impacts of COVID-19 on ECIG use among youth populations. These impacts should be examined further in future studies. This study also had several strengths including using a validated mixed methods approach to identify themes. The sample included ECIG users from across the United States allowing for greater assessment of any potential regional differences. This study was also conducted shortly after COVID-19 stay at home orders were implemented, thus decreasing the issues of recall bias had the study occurred long after the pandemic began as well as when stay at home orders began being lifted.

## 5. Conclusions

COVID-19 has impacted many people around the world; however, this study reveals ECIG users may face additional impacts related to ECIG use and related behaviors. ECIG use may put users at risk for increased exposure to individuals with COVID-19. ECIG use may also contribute to financial burdens that are exacerbated by needing to purchase ECIG supplies due to dependence, stress stemming from addiction, and COVID-19-related health risks that may be increased due to exposure to toxicants in ECIG aerosol [38,39]. Future research should monitor ECIG users over time and the potential long-term effects that COVID-19 may have on ECIG users.

## Figures and Tables

**Table 1:** Sample demographics and ECIG/tobacco use characteristics.

Characteristic	*N*	%
*Age (M, SD)*	(35.1, 10.8)	
*Gender*		
Women	51	54.8
Men	40	43.0
Transgender or other	1	1.1
*Ethnicity*		
Hispanic/Latino(a)	13	14.0
*Race*		
American Indian/Alaskan Native	0	0
Asian	6	6.5
Native Hawaiian/Pacific Islander	0	0
Black/African American	10	10.8
White/European American	71	76.3
More than one race	5	5.4
*Education*		
High School diploma or GED	12	12.9
Some college credit, but less than 1 year	7	7.5
1 or more years of college, no degree	16	17.2
Associate’s degree	11	11.8
Bachelor’s degree	36	38.7
Higher than a bachelor’s degree	10	10.8
*Regular ECIG use history ^1^*		
1–3 months	5	5.4
4–6 months	6	6.5
7–12 months	10	10.8
Between 1–2 years	28	30.1
More than 2 years	43	46.2
*ECIG frequency*		
At least once per day	11	16.3
Every once in a while throughout the day	28	18.4
Fairly frequently throughout the day	38	46.9
Almost always throughout most of the day	14	18.4
*Regular ECIG device*		
Prefilled disposable/Cig-alike	7	18.4
E-hookah	2	2.0
Vape pen/eGo style device	18	16.3
Rebuildable/Mechanical Mod or Box Mod	20	20.4
E-cigar	2	6.1
E-pipe	1	1.1
Pod mod such as JUUL	40	36.7
Disposable vape such as a Posh, Puff bar, Mojo, or other similar device	1	1.1
Other	2	2.2
*ECIG liquid nicotine concentration*		
0 mg/mL	1	1.1
1–4 mg/mL	28	30.1
5–10 mg/mL	28	30.1
11–20 mg/mL	14	15.1
21–30 mg/mL	5	5.4
31–40 mg/mL	2	2.2
50 mg/mL	7	7.5
>50 mg/mL	3	3.2
Don’t know	2	2.2
*Preferred ECIG liquid flavor*		
Menthol or mint	25	26.9
Tobacco	29	31.2
Fruit	23	24.7
Vanilla or crème	3	3.2
Other (including clove, spice, nut, alcoholic drink, coffee/tea, candy, or dessert)	12	12.9
*Location ECIG liquid typically purchased pre-COVID-19 ^2^*		
Vape shop/tobacco store	57	61.3
Gas station or convenience store	29	31.2
Retail store like a grocery store, drug store, or department store	11	11.8
Order online/over the internet	39	41.2
Homemade/Do-it-yourself (DIY)	3	3.2
*ECIG use after waking*		
After 60 minutes	20	21.5
31–60 minutes	30	32.3
6–30 minutes	22	23.7
Within 5 minutes	19	20.4
*E-Cigarette Dependence Scale Score ^3^ (M, SD)*	(2.06, 0.83)	
*E-Cigarette Dependence Scale—Reach for ECIG ^3^*		
Never	2	2.2
Rarely	10	10.8
Sometimes	40	43.0
Often	29	31.2
Almost always	11	11.8
*E-Cigarette Dependence Scale—Vape more before not allowed ^3^*		
Never	5	5.4
Rarely	8	8.6
Sometimes	32	34.4
Often	30	32.3
Almost always	17	18.3
*E-Cigarette Dependence Scale—Drop everything to buy ECIGs ^3^*		
Never	19	20.4
Rarely	31	33.3
Sometimes	20	21.5
Often	18	19.4
Almost always	4	4.3
*E-Cigarette Dependence Scale—Craving gets intolerable ^3^*		
Never	10	10.8
Rarely	23	24.7
Sometimes	37	39.8
Often	11	11.8
Almost always	10	10.8
*Lifetime use of 100+ cigarettes*		
Yes	81	87.1
*Current use of other tobacco products*		
Cigarettes	50	53.8
Cigar	11	11.8
Cigarillo or little cigar	19	20.4
Smokeless	7	7.5
Waterpipe	11	11.8

*Notes*: Total *n* and percentages for sample characteristics is based on the 93 participants who completed the participant questions. ^1^ Regular use was defined as using an ECIG some days or most days. ^2^ Participants could select more than one option for location where they typically purchased their ECIG liquid. ^3^ Items from the 4-item E-Cigarette Dependence Scale [19] including “I find myself reaching for my e-cigarette without thinking about it,” I vape more before going into a situation where vaping is not allowed,” “I drop everything to go out and buy e-cigarettes or e-juice,” and “When I haven’t been able to vape for a few hours, the craving gets intolerable.”.

**Table 2:** ECIG user-identified clusters and statements describing the impacts of COVID-19 on vaping.

Cluster	Statement	Mean Rating
**Stocking Up and Bulk Purchasing ^1^**		**4.13**
	58. I check my supplies to make sure I have what I need.	5.15
	16. I bought extra vaping supplies and e-liquid/pods to stock up.	4.85
	26. I am buying more e-liquid/pods so I don’t have to go to the store as much.	4.31
	12. I order online to make sure I have vaping supplies at home and a steady supply coming in.	4.23
	5. I am purchasing several vaping products at once because I am uncertain if I can leave my home.	4.23
	2. I purchased multiple vaping products because I’m uncertain if vaping products will be available in the future.	4.11
**Challenges in Obtaining ECIG Supplies ^1^**		**3.40**
	9. I go to the store less due to social distancing.	5.54
	73. I am more conscious of prices for vaping products.	4.54
	76. The price of vaping products has increased because of the COVID-19.	4.21
	71. It is challenging to return or replace vaping products.	4.12
	57. I worry that I will not be able to get vaping supplies during the COVID-19 pandemic.	4.06
	15. I am hesitant to go out and get more e-liquid/pods because I am scared to go near other people.	3.63
	19. I am mad that my vape shops are closed and may go out of business due to COVID-19.	3.52
	64. I am low on money and don’t want to have to spend it on vaping products.	3.49
	6. I am worried I may smoke cigarettes because it is difficult to get vaping supplies.	3.10
	4. I have not ordered any new vaping products due to delays caused by COVID-19.	2.79
	13. Ordering vaping products online made me nervous.	2.63
	72. I’ve been using burnt pods because the vape I bought online due to lockdown is defective.	2.46
	46. I am not affected because I make most of my own e-liquid (DIY).	2.46
	74. I have thought about quitting vaping because I bought a defective vape and it takes too long to get a new one.	2.27
	50. Someone makes my e-liquid for me so I don’t have to go to the store.	2.19
	55. I purchase my e-liquid/pods in bulk now.	3.81
	81. I buy e-liquid/pods in bulk to minimize trips to the store, but since I am also vaping more it has balanced out and not really helped.	3.25
	38. I find myself buying more vaping products online because I am bored.	3.21
	
**Alternative Purchasing Procedures ^1^**		**3.27**
	3. I ordered vaping products online.	4.80
	35. I already purchase my vaping products online, so I do not need to go out for them.	4.15
	66. I ordered vaping products/supplies online and paid extra for shipping.	3.77
	68. It is difficult to obtain vaping products in store or online because many things are out of stock.	3.62
	48. I have to call multiple stores to ask if they have the vaping products I want.	3.05
	80. I can only buy vapes/e-cigarettes from a convenience/grocery store so my options are limited.	2.84
	1. I am unable to purchase vaping products.	2.56
	29. I purchased vaping supplies by using curbside pickup at the store.	2.51
	28. I bought a nasty vape/e-cigarette from a convenience store to get through.	2.17
**Increased ECIG Use ^2^**		**4.07**
	43. I like that I can vape while working at home.	4.89
	34. I vape more because I am bored.	4.79
	49. I am trying to vape more than smoke cigarettes.	4.63
	42. I vape more than usual.	4.52
	56. I vape more because I am at home where it is allowed vs. other environments pre-COVID-19.	4.47
	62. I am vaping more because I have to stay home and I am not going other places.	4.32
	17. I am vaping more because I am not currently working and have nothing to do.	3.96
	31. I see my friends less now because of quarantine so I am vaping more to deal with the isolation.	3.89
	45. I vape more because I stay up later and sleep less.	3.80
	53. I try to vape in private because I have increased my usage.	3.31
	36. I vape more often because I am not supposed to smoke cigarettes in the house.	3.16
	21. I vape more because I am around my family more.	3.07
		
**Disruption of Routine and ECIG Use ^2^**		**3.59**
	33. I don’t let anyone else use my vape/e-cigarette.	5.54
	51. I try to distance myself further from people when I vape.	4.70
	32. I have vaped more to help calm my anxiety and the panic feeling caused by COVID-19.	4.30
	59. I crave my vape/e-cigarette more often.	4.28
	75. I am more aware of how much I vape because I have to vape around my family/roommates.	3.79
	47. Vaping has made me stay at home so I avoid contact with others.	3.16
	83. My cravings to vape are becoming unbearable.	3.05
	20. COVID-19 has caused me to smoke more cigarettes.	2.98
	70. I get into arguments with my spouse/partner because they do not like how often I vape.	2.63
	82. My family/roommates won’t let me vape in the house.	2.58
	44. I don’t feel well because I am not able to vape.	2.51
		
**Efforts to Decrease ECIG Use ^2^**		**3.38**
	10. I vape the tanks/pods until the very last drop so I don’t have to go out as often.	4.51
	27. I am trying to ration my e-liquid/pods.	4.06
	67. I am trying to vape less and buy fewer supplies to save money.	3.67
	65. I am more mindful of the amount I vape because it is no longer easy to obtain products in store or online.	3.65
	60. I wait longer to vape after I wake up.	3.48
	30. I find myself going longer in between vaping/not taking as many hits.	3.47
	37. I am vaping less to try to extend what I have instead of buying more online.	3.38
	84. I vape less because I don’t want to go out to buy more supplies.	3.05
	23. I vape less.	2.93
	39. I don’t vape outside anymore.	2.86
	63. I have started rolling my own cigarettes in order to vape and smoke store-bought cigarettes less.	2.09
		
**Improving ECIG Skills ^2^**		**2.90**
	25. I have tried new flavors and brands because of being at home and I don’t have much to do.	3.33
	7. I have more time to perfect my homemade/DIY e-liquid flavors because I am at home more.	2.69
	14. I have learned new vape tricks due to being at home more.	2.68
		
**COVID-19 Health Concerns ^3^**		**3.53**
	24. I worry about how vaping is affecting my health.	4.42
	69. I am concerned about vaping increasing the chances of complications from COVID-19.	3.77
	61. COVID-19 has made me very aware of my chest and lung pain from vaping.	3.70
	8. When I cough while vaping it makes me worried about COVID-19.	3.64
	22. Since I have increased my usage I am afraid of getting popcorn lung.	3.12
	79. I worry about my health so I will stop vaping until COVID-19 is gone for sure.	2.52
**Perceptions of ECIG Use and COVID-19 ^3^**		**3.49**
	54. I am not scared or concerned about vaping due to COVID-19.	3.91
	52. COVID-19 has not affected my vaping at all.	3.88
	78. I have thought about quitting or reducing my vaping because of COVID-19.	3.63
	41. Vaping has made me safe because people keep away from me when I am vaping.	3.37
	77. I’m frustrated because I thought COVID-19 would help me quit, but I am still vaping the same amount.	2.67
**COVID-19 Protection ^3^**		**2.84**
	40. I don’t stress too much because I am pretty healthy.	4.17
	11. I have wondered if vaping provides a protective layer to prevent COVID-19.	2.53
	85. I think vaping may kill the COVID-19 virus due to the heat from vaping.	2.33
	18. I think vaping will increase my immune system.	2.32

*Notes*: Clusters are presented in groups: ^1)^ Purchasing Behaviors and Obtaining ECIG Supplies, ^2)^ Changes in ECIG Use Frequency and Environment, and ^3)^ Health-Related Perceptions. Mean ratings are based on responses to the prompt “This is a way Coronavirus/COVID-19 has impacted my vaping/e-cigarette use, my vaping/e-cigarette related purchasing, or other vaping/e-cigarette related behaviors or issues” using a 7-point scale from 1 (Definitely NOT true for me) to 7 (Definitely true for me).
